# Unintended pregnancy and the factors among currently pregnant married youths in Western Oromia, Ethiopia: A mixed method

**DOI:** 10.1371/journal.pone.0259262

**Published:** 2021-11-04

**Authors:** Jaleta Merga, Desalegn Wirtu, Tariku Tesfaye Bekuma, Misganu Teshoma Regasa

**Affiliations:** 1 Department of Public Health, Institute of Health Sciences, Wollega University, Nekemte, Ethiopia; 2 Department of Midwifery, Institute of Health Sciences, Wollega University, Nekemte, Ethiopia; University of Mississippi Medical Center, UNITED STATES

## Abstract

**Background:**

Unintended pregnancy is a global concern affecting both developed and developing countries. Some young women who had unintended pregnancies obtain an abortion while others carry their pregnancies to term, incurring the risk of morbidity and mortality higher than those for adult women. Despite the availability of highly effective methods of contraception, different studies in Ethiopia revealed that there is a high level of unintended pregnancy.

**Objective:**

To assess the magnitude of unintended pregnancy and associated factors among currently pregnant married youths in Kiremu district.

**Methods:**

A community-based cross-sectional study was conducted among currently pregnant married 15–24 years women. Multi-stage stratified sampling technique was used to select 434 study units. Ten kebeles were randomly selected and samples were selected from each of ten kebeles by simple random sampling using kebeles household identification numbers as the sampling frame. Quantitative data was entered with SPSS version 20 and crude and adjusted odds ratio together with their 95%CI were computed and interpreted accordingly. A p-value<0.05 was considered to declare a result as statistically significant in this study. In-depth interviews and transcripts of the recorded discussions were coded and analyzed thematically. The results were finally presented in texts, tables, and graphs.

**Result:**

Unintended pregnancies among currently pregnant married young women in the study area were 31.1%. Educational status (AOR = 3.195,95%CI = 1.757,5.811),being Gov’t employee (AOR = 0.039, 95% CI = 0.002,0.988), ever heard contraceptives(AOR = 0.260, 95%CI = 0.077, 0.876), ever used contraceptives (AOR = 0.348,95%CI = 0.168,0.717),discussion about contraceptives with husband(AOR = 0.027,95%CI = 0.015, 0.050),fear of side effect of contraceptives (AOR = 5.819,95% CI = 1.438,23.422), autonomy on health (AOR = 0.122,95%CI = 0.035,0.431), age at first marriage (AOR = 3.195, 95%CI = 1.757,5.811), age first pregnancy(AOR = 23.660,95%CI = 12.573,44.522), being visited by health care providers (AOR = 0.202,95%CI = 0.073,0.566) and average birth interval (AOR = 3.472,95%CI = 1.392,8.61) were the factors associated with unintended pregnancy.

**Conclusion and recommendation:**

Significant proportion of women had an unintended pregnancy in the study area. Therefore, emphasis should be given to married youths especially on women empowerment, encouraging partner discussions, and providing appropriate counseling on contraceptive side effects by giving due attention to those marred at younger ages (<18 years).

## Background

Unintended pregnancy is unwanted and unplanned at the time of conception which can further be classified as mistimed and unwanted pregnancy. Mistimed pregnancy occurs in women who wanted a child later on, but they conceived sooner than they had planned, and unwanted pregnancy is defined as the pregnancy happening in women who already have children and do not want any more children. It is a worldwide problem that affects all segments of the population particularly young women and their families due to the negative consequences for both mothers and children [1].

Globally, the maternal mortality ratio (MMR) was 216 maternal deaths per 100,000 live births in 2015 and of the approximately 210 million pregnancies occurring every year, an estimated 40% are unplanned. Developing countries account for approximately 99% of the global maternal deaths in 2015 and of the 182 million pregnancies occurring every year, an estimated 36% are unplanned [2]. Sub-Saharan African countries alone accounting for roughly 66% of the global maternal deaths in 2015 and unintended pregnancy accounts for more than a quarter of the 40 million pregnancies that occur annually among all women of reproductive ages. Of all the unintended pregnancies, 44% occurring among women aged 15–24 years [3]. In Ethiopia, the rate of unintended pregnancy was shown to be lower among women aged 20–24 years than among those aged 15–19 years (22.8% vs. 27.1%, respectively) [4]. The 2016 Ethiopian Demographic and Health Survey (EDHS, 2016) report shows the MMR in Ethiopia was 412 per 100,000 live births and the prevalence of unintended pregnancy was 25% (8% unwanted and 17% mistimed). Many of the mistimed Pregnancies in Ethiopia occurred among women less than 30 years of age and the unmet need for family planning was 22%. According to the report of EDHS, 2016 the prevalence of unintended pregnancy in Oromia was found to be 39%. The majority of these pregnancies arise from the non-use of contraceptive methods among women wishing to avoid or postpone childbearing [5].

Some young women who had unintended pregnancies obtain an abortion. Many of which are performed in unsafe conditions and others carry their pregnancies to term, incurring the risk of morbidity and mortality higher than those for adult women [6]. Young maternal age is associated with a significantly increased risk of maternal anemia, poor prenatal care compliance, preterm delivery, low birth weight, newborn admission to the intensive care unit, and postpartum complications [7]. Many pregnancies occurring in young women are unintended which further increases the risk of problems [8].

Young women face a significant risk of unintended pregnancy because they have less access to, and are less likely to use contraception. If the unmet need related to unintended pregnancy among adolescents was met; 2.1 million unplanned births, 3.2 million abortions, and 5600 maternal deaths could be averted each year [9].

Unintended pregnancy is one of the major reproductive health challenges faced by young women in Ethiopia [10]. According to the Ethiopian Federal Ministry of Health, abortion accounts for 60% of gynecological and almost 30% of all obstetric and gynecological admissions. And over half of 19 million women who annually seek abortions in Ethiopia are between the age of 15–24 years [11].

Studies have shown that the autonomy of women, the communication of partners on family planning, the desire to have children, the travel time to the closest family planning service area, and educational level are significantly associated with unintended pregnancy [12, 13]. The Ethiopian government has developed a national reproductive health strategy that addresses the importance of reducing unwanted pregnancies by increasing the use of contraceptives to 66% which otherwise leads to an estimated 382,000 induced abortions per year [14]. Despite the availability of highly effective contraceptive methods, different studies in Ethiopia have shown that there is a high level of unintended pregnancy [15]. Limited research has described the extent of involuntary pregnancy and associated factors in pregnant women aged 15 to 24 years; even these few studies were conducted in health facilities with mothers receiving antenatal or postnatal care who were themselves affected by pregnancy intention. Existing works of literature also do not provide adequate information on the extent of unintended pregnancy and associated factors among these age groups [16].

Therefore, this study was aimed to determine the prevalence of unintended pregnancy and associated factors among currently pregnant married women aged 15–24 years in Kiremu district, Oromia, Ethiopia. The magnitude and determinants of unintended pregnancy among women aged 15–24 in the Kiremu district have not been clearly understood. The findings of this study are critical in guiding reproductive health program planners, policymakers, and stakeholders to implement effective reproductive health programs that can reduce the risk of maternal morbidity and mortality induced by unintended pregnancies. Information concerning unintended pregnancy levels can point to gaps in access to and uses of contraception. Furthermore, this study can serve as source of information for Kiremu district and for other investigators to develop an action plan.

## Methods

### Study area, period, and design

The study was conducted in Kiremu district East Wollega zone of Oromia regional state of Ethiopia from April to May 2019. Kiremu district is located 473 km from Addis Ababa and 142 km from Nekemte, the capital city of the East Wollega Zone. It is bounded by Amhara regional state in the north, Amuru woreda in the east, Abe dongoro woreda in the south, and Gida Ayana woreda in the west. The district has 19 administrative kebeles (4 urban and 15 rural). The total population of the district was 77151 of which females were 38190 (49.5%) and males were 38961(50.5%) in the year 2019. Of the total of 77151, 3.47% (2677) were pregnant mothers. Updated data from the family folder of health extension workers of the study area shows that there were 704 pregnant women aged 15–24 years in the year 2019. The district has three governmental health centers, 21 health posts and 10 private clinics. The health service coverage of the woreda was 79% in the year 2019.

A community-based cross-sectional study was employed using the quantitative and qualitative method of data collection in Kiremu district.

### Study population

Study population were currently pregnant married women aged 15–24 years from the selected kebeles of Kiremu district whileselected community leaders, Health Developmental Armies and Health Extension Workers were the study populations for the qualitative study.

#### Inclusion criteria

All married pregnant women aged 15–24 years residing in Kiremu district for at least six months before the study period.

#### Exclusion criteria

Pregnant women aged between 15–24 years who were unable to respond and critically ill.

### Sample size determination

The sample size was determined by using single population proportion formula by considering the following assumptions; the prevalence of unintended pregnancy = 23% [17], 95% confidence interval, Z^2^α/2 = 1.96 and a 5% margin of error. Since the total number of pregnant women in the district was less than 10,000, a correction formula was used to come up with the final sample size.


$$\frac{ni={\left(z_{\alpha /2}\right)}^{2}.P(1‐P)}{{\left(d\right)}^{2}}=\frac{{\left(1.96\right)}^{2}.0.23(1‐0.23)}{{\left(0.05\right)}^{2}}$$

The calculated sample size = 272

n_f = n/1+n/N = 272/1+272/704_

= 197

Considering design effect of 2 and adding non response rate of 10% the final sample size was:

197x2 = 394+40 = 434

In-depth interview was conducted with six key informants in the selected kebeles.

### Sampling technique and selection procedure

**Fig 1:**
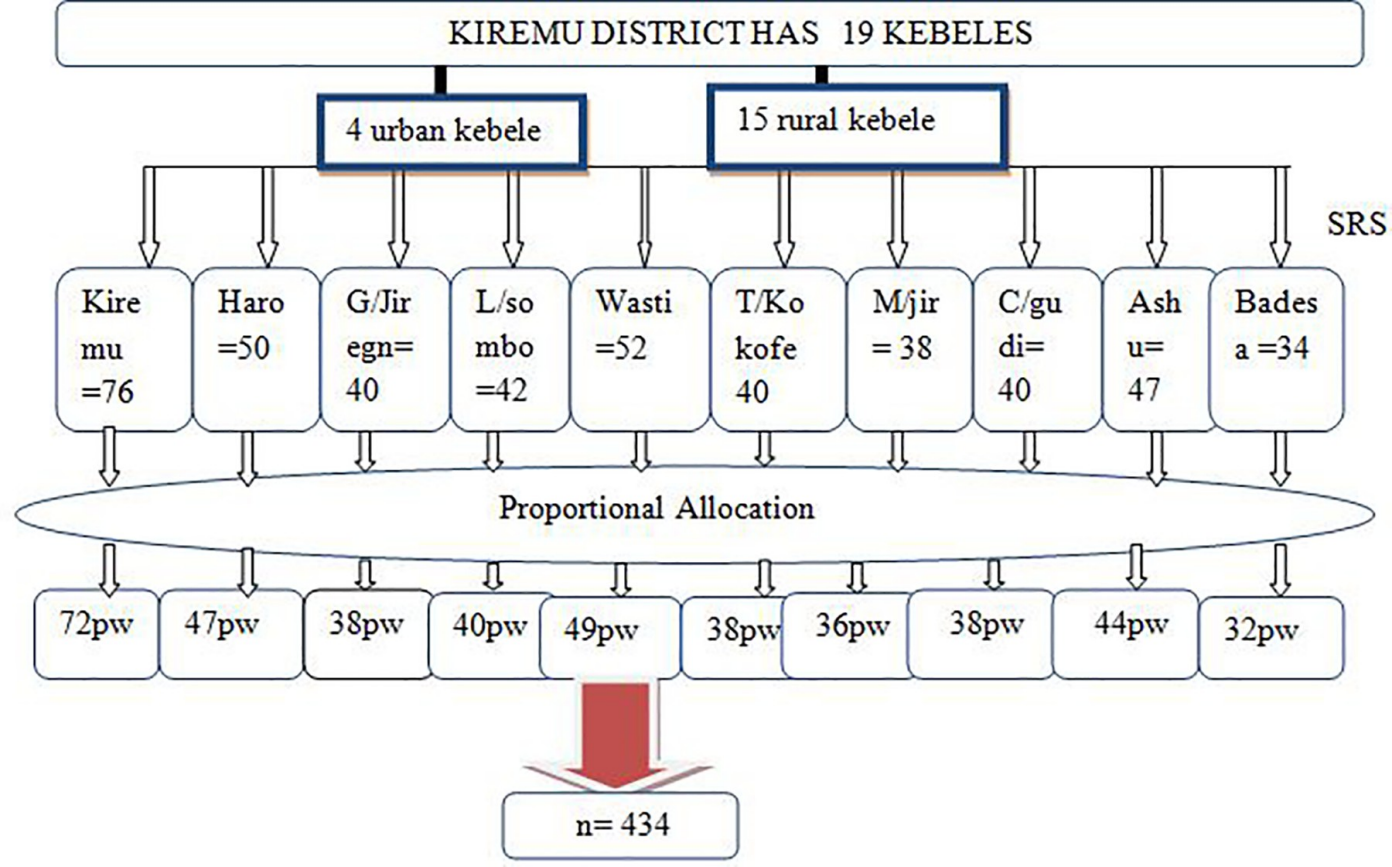


Kiremu district has 19 administrative kebeles. According to updated data from the family folder of health extension workers there were704 pregnant women aged 15–24 years in the district. A multistage stratified sampling technique was used to select 434 currently pregnant married women aged 15–24 years women from the district. First, the 19 kebeles in the district were stratified into two (15 rural kebeles and 4 urban kebele) and 10 kebeles (two from urban and eight from rural) were selected by simple random sampling method and from the selected kebeles, households with eligible women were identified and listed from the family folder after which the households in which eligible woman resides were selected using simple random sampling. Whenever more than one eligible respondent found in the selected households, only one respondent was selected by using lottery method [**Fig 1**].

For qualitative data participants were selected purposively, based on their knowledge or who knows what is going in the community for an interview.

### Data collection tools and procedure

Quantitative data was collected using an interviewer-based structured questionnaire with closed-ended questions that were adapted from different reviewed literatures. It consists of socio-demographic, economic, cultural, reproductive and health service-related factors. Two days of training was given to data collectors and supervisors on the questionnaire, approach to the interviewees, interviewing techniques, privacy, and confidentiality of the respondents. Data was collected by 12 trained Health extension workers and supervised by 3 trained BSc. public health professionals. Qualitative data was collected by using an unstructured questionnaire and an in-depth interview was conducted with 2 Community leaders, 2 members of health development armies, and 2 Health extension workers in Kiremu-01, Wadesa Dima, Haro-01, and Lalistu Sombo kebeles concerning associated factors of unintended pregnancy.

### Study variables

#### Dependent variables

Unintended Pregnancy

#### Independent variables

*Socio-demographic and economic characteristics*. Age, Occupation, Level of education, Place of residence.

*Socio-cultural factors*. Spousal communication, Women’s autonomy, Taboos, and influence of other important families.

*Reproductive factors*. Age at first pregnancy, age at first marriage, Birth interval, Gravidity, Parity, child preference.

*Health service-related factors*. Accessibility and Availability of FP methods, Knowledge and practice of FP methods, Fear of side effects and health concerns, visiting health care providers, travel time from home to health facilities.

#### Operational definition

*Youths*: *Young women between the age of 15–24 years*. *Ever use of contraceptives*. Respondents’ use of contraceptives at least once in her lifetime (15).

*Awareness*. defined as awareness of any method of modern contraception (13)

*Women’s autonomy*. Refers to a woman’ control over resources, and her ability to make decisions on her own and to act upon these decisions (17). In this study, it’s to mean women decision-making power on their health care which was analyzed from the question which asks about the person who usually decides on respondent’s health care, followed by 5 responses: the respondent herself, her husband/partner, husband’s father, husband’s mother and others.

*Accessibility*. Is the extent to which an appropriate package of contraceptive methods can be obtained by mothers in the study area.

*Appropriate package*. Provision of contraceptive methods along with instructions about correct and consistent use, help the client develops a plan for using the selected method and for follow-up, confirm client understanding and allow time for addressing questions.

*Kebele*. the lowest government administrative hierarchy that exists next to woreda.

### Data processing and analysis

Data were entered using Epi info version 7.0 and analyzed using Statistical Package for social scientist (SPSS) Software Version 20. Analyses were done through univariate, bivariate, and multivariate analysis. Proportions and percentages were calculated to show the distribution of the respondents by socioeconomic, demographic, cultural, health service-related and reproductive related factors. All explanatory variables that have an association in bivariate analysis with a p-value less than 0.25 were entered into the multivariable logistic regression model. Odds ratio with 95% confidence interval was computed to assess the presence and degree of association between dependent and independent variables and the level of significance of association is determined at P-value<0.05. The tables, texts, and charts were used to present the results. Data received through key informant interviews were recorded using a recorder and transcribed to text files. Transcript of the recorded discussions was coded and analyzed using thematically. Findings from the quantitative part were triangulated with the qualitative results. In fitting multiple regression models, the first thing to be done is to examine the existence of inter-correlation among explanatory variables. The existence of this effect in the models can be checked by using tolerance or variance inflation factor (VIF). Tolerance is 1- R^2^ for the regression of that independent variable on the other independents, ignoring the dependent.

The higher the intercorrelation of the independents, the more the tolerance will approach zero. As a rule of thumb, if tolerance is less than 0.20, a problem with multicollinearity is indicated. VIF > = 4 suggests a multicollinearity problem. The goodness of fit of the models was assessed by using the Hosmer and Lemeshow goodness of fit test and insignificant value of test was taken as the goodness fit of the model.

### Data quality management

The tool was developed as per the specific objectives of the study and checked whether the respective questions were designed in a way the specific research questions are answered. Since the tool was adopted from previous studies like EDHS [5, 18], no further validation was done except pre-test which was done on 5% of the total sample size with the involvement of all research teams in Kokofe-01 kebele and wording, translations, and time necessary for an interview were modified.

English version questionnaire was translated into Afan Oromo and then back to English to ensure its consistency with great emphasis given to local vocabularies. The principal investigator and supervisor gave feedback and correction on daily basis for the data collectors before they deployed to the field and completeness, accuracy, and clarity of the collected data was checked carefully. Any errors, ambiguity, incompleteness encountered were addressed on the following day before starting the next day activities.

### Ethics approval

Ethical clearance for the study was obtained from the Research ethics committee of the institute of health sciences, Wollega University. Formal letters were written to all concerned authorities and permission was secured at all levels. Informed verbal consent was obtained from each respondent after explaining the purpose and procedure of the study. No name or other identifying information was included in the data collection tool. Considering the sensitivity of this research, all the basic principles of human research ethics (respect of persons, beneficence, voluntary participation, confidentiality, and justice) have been respected.

## Results

### Socio-demographic characteristics of the study subjects

From the total 434 calculated sample, 424 were interviewed making a response rate of 97.6%. Three hundred, (70.8%), were from rural and 124 (29.2%) were from urban areas of the district. Most of the study subjects, 360 (84.9%), were between the age of 20–24 years followed by 15–19 age groups (64)15.1%. Regarding their religion, 151(35.6%), were Orthodox Christian and 151(35.6%), were protestant religious followers. Concerning the ethnicity of the respondent, the majority, 330(77.8%), were Oromo, followed by Amhara, 94(22.2%) [**Table 1**].

**Table 1:** Socio-demographic characteristics of currently pregnant married women youths of Kiremu district, Oromia, Ethiopia, 2020.

Variables		Frequency (%)
**Residence**	Urban	124 (29.2)
	Rural	300 (70.8)
**Age**	15–19	64 (15.1)
	20–24	360 (84.9)
**Religion**	Orthodox	151 (35.1)
	Muslim	121 (28.5)
	Protestant	151 (35.1)
	Wakefata	1 (0.20)
**Ethnicity**	Oromo	330 (77.8)
	Amhara	94 (22.2)
**Educational status**	Cannot read and write	179 (42.2)
	1–4	65 (15.3)
	5–8	70 (16.5)
	9–12	64 (15.1)
	College and above	46 (10.8)
**Occupation of respondents**	Student	59 (13.9)
	Farmer	204 (48.1)
	Government employed	32 (17.5)
	Daily laborer	89 (21.0)
	Unemployed	34 (8.0)
	Merchant	6 (1.5)
**Occupation of partners**	Student	55 (13.0)
	Farmer	217 (51.0)
	Government employed	57 (13.4)
	Daily laborer	74 (17.5)
	Unemployed	13 (3.10)
	Merchant	8 (1.90)

### Socio-cultural characteristics of the study subjects

**Fig 2:**
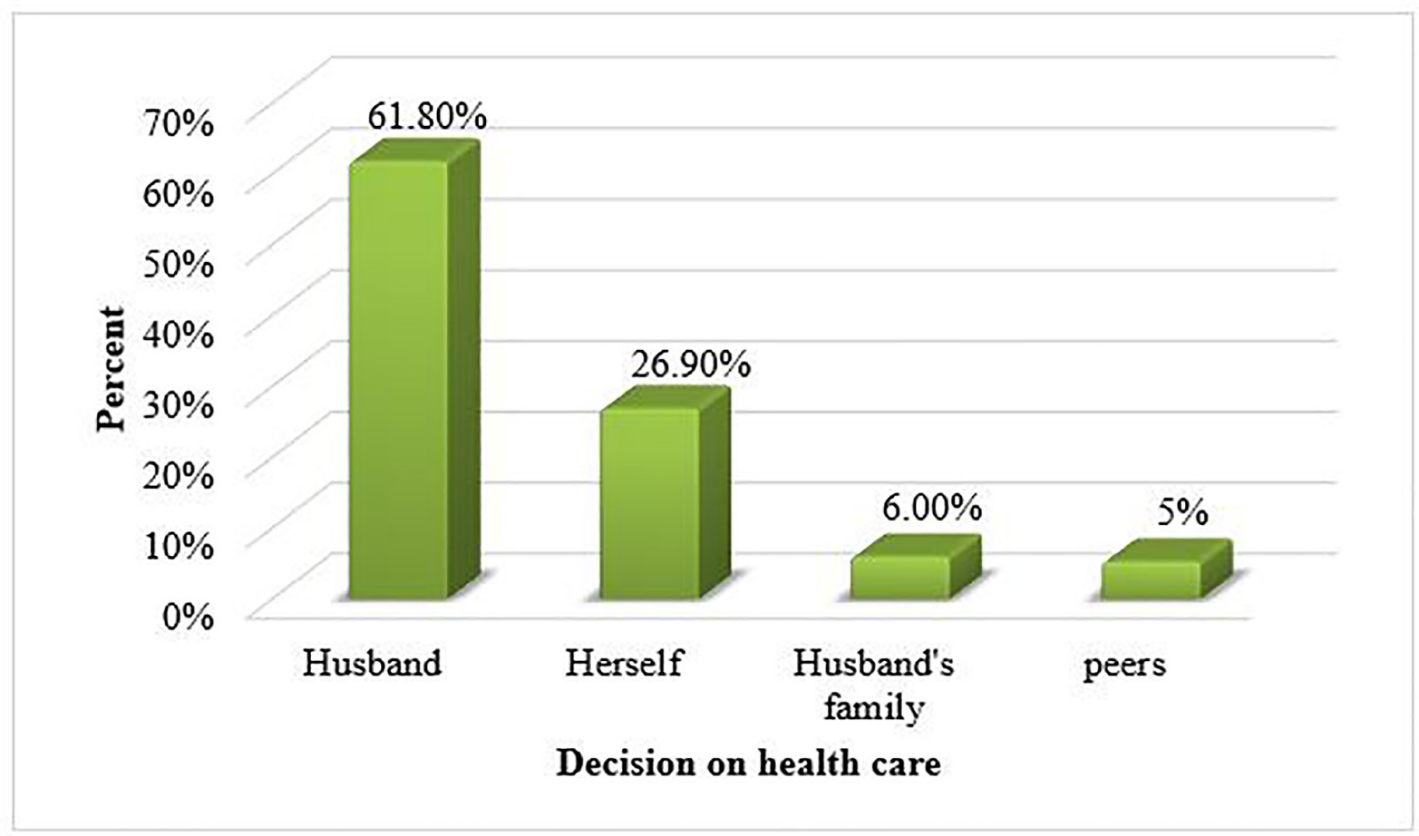


The majority, 262(61.8%) of the respondents decided to seek health care by their husband and followed by themselves, 114(26.9%), Husband’s family 27(6.4%) and peers 21(5.3%) [**Fig 2**].

Nearly all respondents, 405 (95.5%) have ever heard about contraceptives. Concerning information sources for modern contraceptives more than three fourth of respondents, 323(79.8%) reported health extension workers as the source of information for modern contraceptives that was followed by friends, 90 (22.2%) and mass media, 64 (15.8%). Nearly all respondents, 405 (95.5%) know the place to access modern contraceptives. More than one-third of respondents, 151(35.6%) reported having access to health facilities within 30–60 minutes on foot while 123(29.0%) access the health facilities within < 30 minutes from their residence [**Table 2**].

**Table 2:** Source of information and place to access modern contraceptives of currently pregnant married women youths of Kiremu district, Oromia, Ethiopia, 2020.

*Variables*		*Frequency (%)*
***Ever heard contraception(n = 424)***		
	Yes	405 (95.5)
	No	19 (4.5)
***Information source on contraception***		
	Husband	21 (5.5)
	Friends	90 (22.2)
	Health facilities	323 (79.8)
	School	29 (7.2)
	Mass media (TV, Radio)	64 (15.8)
	Others (Relatives)	9 (2.2)
***Know the place of contraception***		
	Yes	405 (95.5)
	No	19 (4.5)
***Time it takes from home to facility***		
	<30min	123 (29)
	30-60min	151 (35.6)
	>60min	150 (35.4)

Regarding awareness of specific family planning methods, more than three- quarter of the respondents, 345(85.2%), knew injectable followed by implants,176 (43.5%), pills, 93 (23.0%), IUCD, 57 (14.1%), sterilization,13 (3.2%). Regarding utilization of family planning methods, 326(80.5%), of the mothers have ever used family planning services; while the rest, 79(19.5%) did not. The main reasons for not using contraceptive methods were fear of side effects, 36(45.6%), and husbands desire more children, 34(43.1%) [**Table 3**].

**Table 3:** Awareness and practice of modern contraceptives of currently pregnant married women aged 15–24 years of Kiremu district, Oromia, Ethiopia, 2020.

Variables		Frequency (%)
**Awareness of specific family planning methods**	Injectables	345 (85.2)
	Pills	93 (93.0)
	Implants	176 (43.5)
	IUCD	57 (14.1)
	Sterilization	13 (3.2)
	Other(condom)	4 (1.0)
**Ever used contraceptives**	Yes	326 (80.5)
	No	79 (19.5)
**Reasons for not using contraceptives**	Husband desiring more children	34 (45.6)
	Fear of side effect	36 (45.6)
	Religious reasons	20 (25.3)
	Lack of knowledge of FP	20 (25.3)
	Husband opposition	26 (32.9)
	Peers/Relatives	25 (31.6)

### Reproductive history

Of the total respondents, 299(70.5%) have ever experienced pregnancy while 125(29.5%) had not. Three hundred two, (71.2%) got pregnant for the first time at the age of 18years and above while, 122(28.8%) were pregnant for the first time at the age of less than18 years. Two hundred sixty-one, (87.3%) respondents have a birth interval of less than 2 years. Forty-nine, (16.4%) of respondents had a previous history of abortion. More than half of the abortions, (61.2%) were spontaneous and from these abortion cases, 21 (51.0%) received abortion care services from government facilities [**Table 4**].

**Table 4:** Reproductive characteristics of currently pregnant married women youths of Kiremu district, Oromia, Ethiopia, 2020.

Variables		Frequency (%)
**Ever been pregnant (n = 424)**	Yes	299 (70.5)
	No	125 (29.5)
**Age first pregnancy(n = 424)**	<18 years	122 (28.8)
	> = 18 years	302 (71.2)
**Age at first marriage(n = 424)**	<18 years	293 (69.1)
	> = 18 years	131 (30.9)
**Average interval between births(n = 299)**	<2 years	261 (87.3)
	> = 2 years	38 (12.7)
**Ever experienced abortion (n = 299)**	Yes	49 (16.4)
	No	250 (83.6)
**Types of abortion (n = 49)**	Induced	19 (38.8)
	Spontaneous	30 (61.2)
**Place of abortion(n = 49)**	Public facility	25 (51.0)
	Private clinic	9 (18.4)
	Pharmacy	2 (4.1)
	Traditional healers	13 (26.1)

#### The current pregnancy status

**Fig 3:**
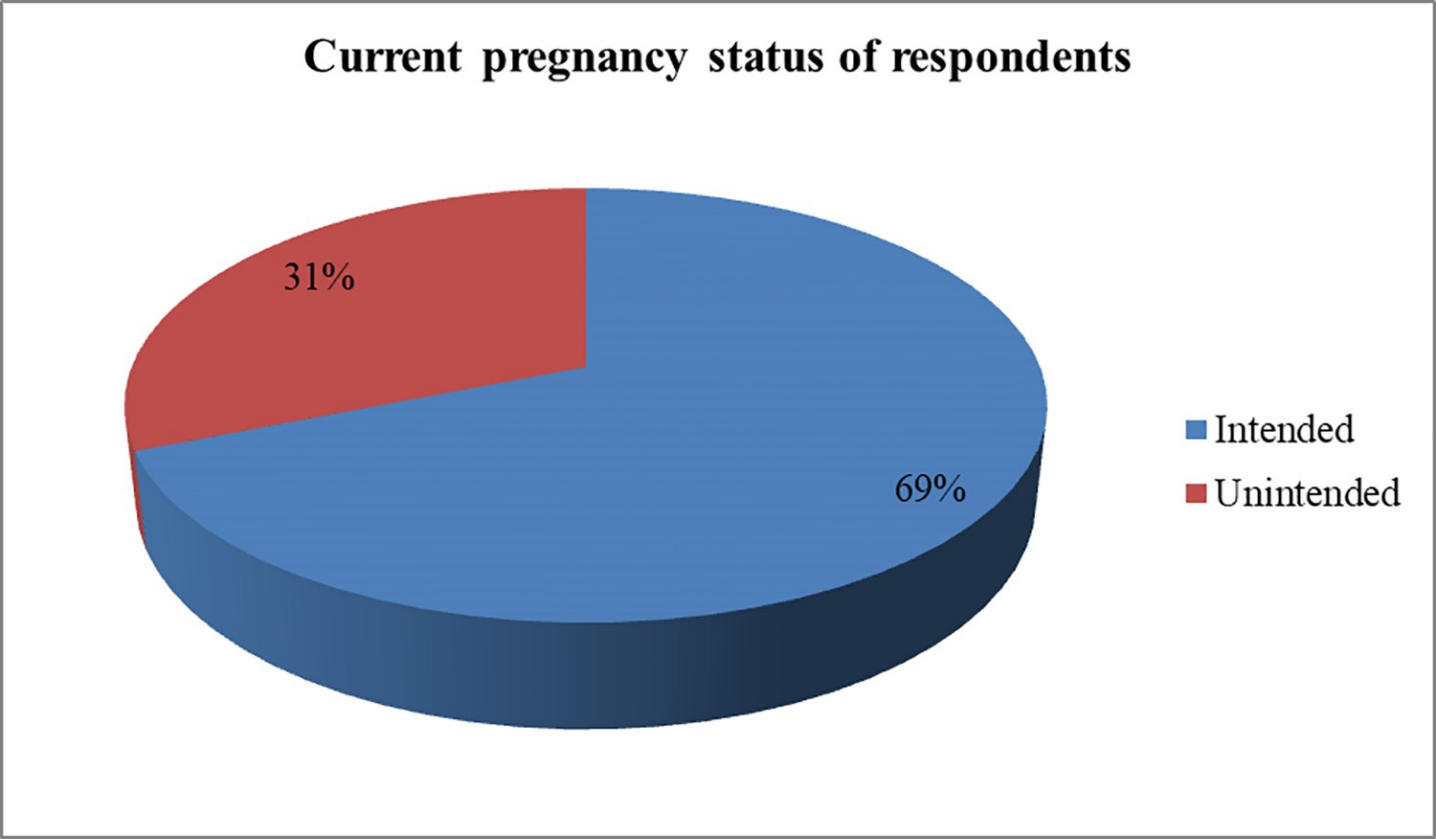


More than two-third, 292 (68.9%) and about one-third, 132 (31.1%), of the current pregnancies were intended and unintended respectively [**Fig 3**].

**Fig 4:**
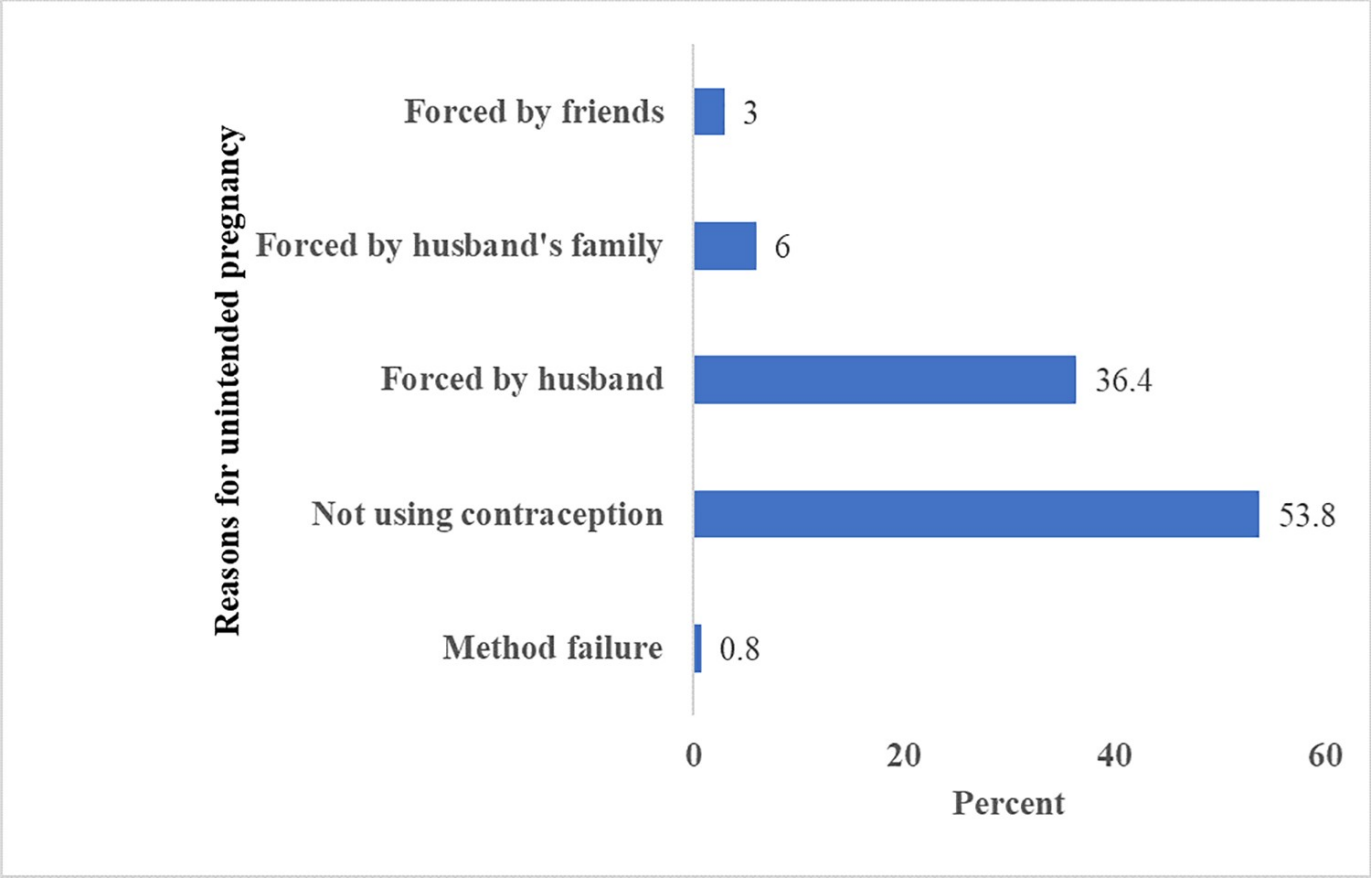


The most frequently mentioned reasons, why they currently experienced unintended pregnancy were not using contraception 71(53.8%), forced by the husband to be pregnant 48(36.4%) followed by forced by husband’s family 8 (6.0%) and peers 4(3.0%) [**Fig 4**].

#### Factors associated with unintended pregnancies

On the bivariate analysis, age of respondents, educational status of respondents, occupation of respondents, Using electronic media, Decision maker on respondents health care, ever heard contraceptives, discussion with husband to use contraceptives, not using contraceptives due to fear of side effect, not using contraceptives due to religious reasons, being visited by health providers, Average interval between births, Age at first pregnancy, age at first marriage was identified to be significantly associated with unintended pregnancy. However, only educational status of respondents, educational status of partners, Occupation of respondents, Occupation of partners, discussion with husband to use contraceptives, Decision on health care, not using contraceptives due to fear of side effect, being visited by health providers, Average interval between births, Age at first pregnancy, age at first marriage were remained significantly associated with unintended pregnancy at the multiple logistic regression analysis.

Currently married pregnant young women who were illiterate are 3 times more likely to have an unintended pregnancy than those who were literate. (AOR = 3, 95%CI = 1.614, 6. 055).

Concerning age at first marriage, pregnant mothers who had a marriage before 18 years old were 3 times (AOR = 3.195, 95% CI 1.757, 5.811) at risk of unintended pregnancy than pregnant women who married at age 18 and above. The occupational status of respondents is negatively associated with unintended pregnancy. Women that become government employee were 96% times less likely to face unintended pregnancy (AOR = 0.039, 95% CI: 0.002, 0.988). Those women who decided on their health care by themselves were 88% times less likely to have an unintended pregnancy as compared to those decisions made by others (her family) (AOR = 0.122, 95%CI: 0.035, 0.431).

Discussion with husband about modern contraceptive methods is also negatively associated with unintended pregnancy. Those women who discuss about contraceptives with their husband were 97% times less likely to have an unintended pregnancy as compared to those didn’t discuss (AOR = 0.027, 95%CI: 0.035, 0.431). Ever heard modern contraceptive methods are negatively associated with unintended pregnancy. Those women who ever heard about contraceptives were 74% times less likely to have an unintended pregnancy than those who never heard (AOR = 0.260; 95% CI: 0.077, 0.876). Women that used any type of contraceptive method before were 65% times less likely to face an unplanned pregnancy, when compared with women that no used contraceptives before (AOR = 0.348, 95% CI: 0.168, 0.717). Not using contraception due to fear of side effects is positively associated with unintended pregnancy. Women who were not using modern contraceptive methods due to fear of side effects were almost 8 times more likely to have an unintended pregnancy as compared to women who did not fear of side effects (AOR = 7.868, 95%CI: 1.767, 35.028). Age of women at first pregnancy is also positively associated with unintended pregnancy. Women who had their first pregnancy below 18 years were 23 times more likely to experience unintended pregnancy when compared to women who had their first pregnancy at 18 years and above (AOR = 23.660, 95%CI: 12.573, 44.522). The interval between births is also strongly associated with unintended pregnancy. Those women who have an average birth interval of below 2 years were almost 3 times more likely to have unintended pregnancy when compared to women who have an average birth interval of 2 years and above (AOR = 3.472, 95%CI:1.392,8.661).

Being visited by health workers for any service is also negatively associated with unintended pregnancy. Those respondents who had been visited by health workers for any service were 88% times less likely to experience unintended pregnancy when compared to those who had not been visited by health workers (AOR = 0.122, 95%CI: 0.061, 0.242) [**Table 5**].

**Table 5:** Factors associated with unintended pregnancy among youths currently married pregnant women of Kiremu district, Oromia Ethiopia, 2020.

Variables	Unintended pregnancy		COR (95% CI)	AOR (95%CI)	P-value	
	Yes (%)	No (%)				
**Educational status of Respondents**					
No formal education	77(43)	102(57)	**2.608(1.711,3.975)**	**3.126(1.614,3.975)**	0.001	
Have formal education	55(22.4)	190(77.6)	1.00	1.00		
**Occupation of respondents**					
Student	42(71.2)	59(28.9)	2.471(0.453,13.478)	0.271(0.021,3.455)	0.315
Farmer	145(71.1)	59(28.9)	2.458(0.482,12.527)	0.229(0.020,3.455)	0.235
Government employed	31(96.9)	1(3.1)	**31.00(2.409,398.879)**	**0.039(0.002,0.988)**	*0.049
Daily laborer	58(65.2)	31(34.8)	1.871(0.356,9.827)	0.46(0.032,3.720)	0.381
Unemployed	13(38.2)	21(61.8)	0.619(0.108,3.539)	0.948(0.095,9.497)	0.964
Merchant	3(50)	3(50)	1.00	1.00	
**Using electronic media**					
Yes	48(23.9)	153(76.1)	0.519(**0.340,0.792)**	0.631(0.269,1.480)	0.290
No	84(37.7)	139(62.3)	1.00	1.00	
**Decision maker on respondents’ health care**					
Her self	103(90.4)	11(9.6)	**5.762(1.961,16.935)**	**0.122(0.035,0.431)**	*0.001
Husband	160(61.1)	102(38.9)	0.965(0.387,2.410)	0.741(0.246,2.231)	0.594
Husband’s father	3(50)	3(50)	0.615(0.099,3.823)	0.955(0.089,10.301)	0.970
Husband’s mother	13(61.9)	8(38.1)	1.000(0.288,3.475)	0.548(0.123,2.447)	0.430
Her family	13(61.9)	8(38.1)	1.00	1.00	
**Discussion with husband on FP**					
Yes	39(13.4)	253(86.6)	**0.028(0.015,0.049)**	**0.027(0.035,0.431)**	*0.001
No	112(84.4)	20(15.2)	1.00	1.00	
**Ever heard FP**					
Yes	121(29.9)	284(70.1)	**0.310(0.122,0.789)**	**0.260(0.077,0.876)**	*0.030
No	11(57.9)	8(42.1)	1.00	1.00	
**Ever used any FP**					
Yes	75(23)	251(77)	0.226(0.135,0.378)	**0.348(0.168,0.717)**	*0.004
No	45(57)	34(43)	1.00	1.00	
**Fear of FP side effect**					
Yes	30(83.3)	6(16.7)	**9.333(3.177,27.422)**	**7.868(1.767,35.028)**	*0.007
No	15(34.9)	28(65.1)	1.00	1.00	
**Age at first marriage**					
<18 years	110(37.7)	183(62.5)	**2.978(1.779,4.987)**	**3.195(1.757,5.811)**	*0.000
> = 18 years	22(16.8)	109(83.2)	1.00	1.00	
**Age first pregnancy**					
<18 years	94(71.2)	28(9.6)	**23.323(13.565, 40.101)**	**23.660(12.573,44.522)**	*0.000
> = 18 years	38(28.8)	264(90.4)	1.00	1.00	

## Discussions

This study has assessed the prevalence and associated factors of unintended pregnancy among currently pregnant married 15–24 years women in Kiremu district, East Wollega, Oromia, Ethiopia. Accordingly, 132 (31.1%) of current pregnancies were unintended. From these unintended pregnancies, 116 (87.9%) of them were mistimed and 16 (12.1%) were unwanted. In contrary to this study results, in unintended pregnancy were 69.8% Adigrat of Tigray whereas 10.27% in west Belessa district [19, 20], in Jimma town 27% were unintended [16], Gondar town 38.9% were unintended, Dessie city 26.7% were unintended [21], west Wollega, Ganji district 23% were unintended [17]. These might be due to the differences in socio-cultural characteristics, health, and geographical coverage. Differences in the availability, accessibility, and quality of health care of maternal health services, including access to modern contraceptives as well as health care provider’s commitment among study area contribute to the differences.

This study shows that the possibility of having unintended pregnancy of currently pregnant married women decreased with the literacy of the women. Currently pregnant married 15–24 years women who cannot read and write are greater than three times more likely to have an unintended pregnancy than those who were literate (AOR = 3 at 95%CI (1.614,6.055). Likewise, a study done in Hawassa, SNNPR indicated that the odds of unplanned pregnancy were higher by 4.6 times among illiterate compared to diploma and above educational Level [22]. Similarly, study conducted in Bale zone among married women showed that women that had an educational level of 10+, certificate, and above were also less likely to encounter an unplanned pregnancy [23]. This may be as a result of educated women is more likely to know about contraceptive methods and to be more convinced in approaching service providers than women with no education. Moreover, it affects positively women’s attitudes towards contraceptive use and puts them in a position to negotiate contraception adoption.

In this study, age at first marriage before18 years was positively associated with unintended pregnancy as compared to age at first marriage at 18 years and above years which is similar to the study conducted in Ganji and Nepal [17, 24]. This could be due to the problem of negotiation power they are more likely to be influenced by their husbands, husband’s family, and norms in the community, and they were less likely in seeking reproductive health services for the reduction of unintended pregnancy. It can be also explained by the fact that in most instances women at this age have not sufficient knowledge for contraceptive utilization, and they were ashamed to take reproductive health services at a younger age.

The occupation was also shown to be another important variable significantly associated with unintended pregnancy. Those who became government employees were less likely when compared to merchants. This result is in agreement with the result of the study done in Bale zone where occupation was found to be a determinant of a woman experiencing unintended pregnancy [23]. The possible justification for this might be government employees are more educated and as the educational level increases the negotiation power with their husband and the confidence in seeking and interacting with the service provider also increased so the use of modern contraceptives increases. Also, they have no time to care for children as they are working outside the home and hence, they are forced to use FP.

Awareness of contraceptives was significantly associated with unintended pregnancy. In this regard, those who ever heard about modern contraceptives were less likely to have an unintended pregnancy than their counterparts (AOR = 0.260; CI95%, 0.077, 0.876). This finding is also in line with findings in Ganji and Senegal [25, 26]. This implies the importance of increasing awareness of the women on family planning, as an important strategy in tackling unintended pregnancy and its consequences.

Contraceptive utilization history has been associated with unintended pregnancy. In this aspect, a 65% reduction of unintended pregnancy was observed in women who had a history of family planning utilization as compared to women who had no history of family planning utilization. It is in line with the study conducted in Hawassa, Debre Markos, and Gamo Gofa [22, 27, 28]. This may happen due to the effect of family planning utilization on the number of children a woman wishes to have, including the choice to have no children, as well as the age at which she wishes to have them.

Discussion about FP with partners in the past 12months before this study was found to be negatively associated with unintended pregnancy. Those currently married 15–24 years women who had discussed about FP issue with their partners were by 97% less likely to have an unintended pregnancy when compared to those who had not discussed it (AOR = 0.027, 95%CI: 0.015, 0.050).

*One of the key informant interviews said that*: *“Most couples in our community don’t discuss when they need to have child*, *on contraception uses to arrive in the decision”*. *(Community leader*, *age-34*, *male)*. *Another key informant interview supports this idea*: *“Couples don’t discuss about the time when they have to have a child and not being pregnant before they have enough money*.*” (Community leader*, *age- 46*, *male)*. The result is consistent with a study done in Debre-Markos town showed that women who do not discuss about modern contraceptives with their husband were 4.38 times more likely to report their current pregnancy unintended than their counterparts [27]. This might be because couples’ communication (discussion of currently married women with their partners) on matters concerning FP and reproductive health provides an enabling environment for mothers to apply their fertility desires and contraceptive needs. This may show the importance of male involvement in the FP program and enhancing spousal discussion to decrease the unmet need for FP.

The study suggests the possibility of having unintended pregnancies decreased as the decision-making power of women increased. The Decision-making power of the women is found to be negatively associated with unintended pregnancy. Those currently married 15–24 years women who have decision-making power less likely to have their current pregnancy unintended when compared to those who have no decision-making power (AOR = 0.122, 95%CI: 0.035, 0.431). *One of the key informant interviews said that*: “*I wanted to take modern contraception consecutively*. *But my husband did not allow me to take the contraception*. *He was against using any method”*. *(Health Development Army*, *35 years*, *female)*. *Another key informant interview raised a similar idea*: *“most young women in our community become pregnant because of their husbands’ influence/ their husbands want to have more children” (Health Development Army*, *40years*, *female)*.

This is in line with the study conducted in developing countries, in which women’s autonomy is negatively associated with unintended pregnancy [29]. Similarly, study conducted in Bangladesh, shows an increase in the autonomy scale decreases the odds of unintended pregnancy by 16% [30]. This is because women are given more power to decide for themselves, on household conditions, when and where to seek reproductive health care.

This study shows that the likelihood of having unintended pregnancy for currently married women decreased with increased being visited by healthcare providers (HEW/HW). Women who have not visited by healthcare providers before this pregnancy were more likely to have an unintended pregnancy than those who have not visited. The result is consistent with the study conducted in Debre Markos town, in which women who were not visited by health extension workers are 3 times more likely to report unintended pregnancy when compared to those who were visited by health extension workers [27]. This is related to their role in providing women with knowledge on family planning, find ways to deal with side effects, and increasing the current use of contraceptives which may result in a low percentage of unintended pregnancy.

Age at first pregnancy is also strongly associated with unintended pregnancy (AOR = 23.660, 95%CI: 12.573, 44.522), which signifies women who were their age at first pregnancy <18 years were almost 24 times more likely to have an unintended pregnancy than their counterparts. This is in alignment with the study conducted in North West Ethiopia, Gondar town in which respondents who were their age at first pregnancy < 18 years were more likely to be unintended pregnancy than who’s their age at first pregnancy ≥ 18 years [31]. The explanation could be the fact that awareness and level of decision-making increase with age and those young women were ashamed to come to the health center to get reproductive health care. And also, those young women are not allowed by their partner and family to utilize contraception due to different reasons raised by the culture.

Fear of side effects of modern contraceptives is also strongly associated with unintended pregnancy. A woman who responds as if they fear of modern contraceptives’ side effects was almost 8 times more likely to experience an unintended pregnancy. (AOR = 7.868, 95%CI: 1.767, 35.028). *One of the key informant interviews said that*: *“most women didn’t use modern contraceptive methods because it causes health problems like increasing their weight and blood pressure*.*” (Health Development Army*, *29 years*, *female)*. *Another key informant interview said that*: *“most women in our kebele didn’t use modern contraceptive methods because if we use the method the drug causes health problems like weight changes*.*” (Health Development Army*, *38years*, *female)*. This is in agreement with the study conducted in the Amhara region, Dessie city in which women who reported a history of side effects while using contraceptive methods were almost 8 times more likely to have an unintended pregnancy as compared to women with no history of side effect [0]. [AOR = 7.92, 95% CI: 3.05, 20.56]. The possible reason could be those women complaining of contraceptive side effects may not use contraceptive methods properly that they may not follow the instruction given by service providers or they may interrupt to use the given method so that they will have more chance to get unintended pregnancy. The interval between births is also significantly associated with unintended pregnancy. Those women whose interval between births <2 years were almost 3 times more likely to experience an unintended pregnancy. (AOR = 3.472, 95%CI: 1.392, 8.661). It is supported by a study done in Pakistan in which those women with short birth intervals were high likelihood of experiencing unintended pregnancy than their counterparts [32]. This might be due to gaps in counseling and postpartum contraception and increased unmet need which in turn results in the high likelihood of having an unintended pregnancy.

### Strength of the study

In this study, qualitative and quantitative methods improve the research outcomes as the qualitative part complement and strengthen the quantitative study. The use female data collectors as women openly discuss reproductive matters with women than men.

### Limitation of the study

Age related responses are prone to recall bias. The other possible limitations are cross-sectional nature of the study which is inability to show cause an effect relationship. The study also included only married pregnant women which cannot be generalized for unmarried youths among whom the experiences of unintended pregnancies might be more common.

## Conclusions and recommendations

The findings of this study indicated that nearly one-third of the study women had unintended pregnancies which is found to be higher compared with other studies. The main reasons stated by study participants for unintended pregnancies were not using contraception, forced by the husband, and forced by the husband’s mother. Level of education, decision-making power in household, age at first marriage, occupational status of respondents, discussing with partners about family planning, o, fear of contraceptive side effects, ever heard and use contraception, and being visited by health providers for any other services were the factors those significantly associated with unintended pregnancy. Therefore, emphasis should be given to married youths especially on women empowerment, encouraging partner discussions, and providing appropriate counseling on contraceptive side effects by giving due attention to those marred at younger ages (<18 years). further in- large-scale study is recommended to explore the underlying causes of unintended pregnancy among married youths.

## Supporting information

S1 File Study questionnaire English and Afan Oromo version. (PDF)

S2 File Key informant interview guide English and Afan Oromo version. (PDF)

S1 Dataset Data in SPSS format. (SAV)

## List of abbreviations and acronyms

AIDS: Acquired Immunodeficiency Syndrome
CDC: Communicable Disease Control
CHIS: Community Health Information System
CSA: Central Statistical Agency
DC: Data Collector
EC: Emergency Contraceptive
EDHS: Ethiopian Demographic Health Survey
FMOH: Federal Ministry of Health
FP: Family Planning
HIV: Human Immunodeficiency Virus
IRB: Institutional Review Board
IUCD: Intrauterine Contraceptive Device
MMR: Maternal Mortality Ratio
SPSS: Statistical Package for Social Science
UNFPA: United Nation Family Planning Agency
UNICEF: United Nation International Children’s Emergency Fund
WHO: World Health Organization
PI: Principal Investigator
PSU: Primary Sampling Unit
PRB: Population Reference Bureau
PW: Pregnant Women
SRH: Sexual and Reproductive Health
SSU: Secondary Sampling Unit
