# Supplementary material for: Unintended pregnancy and the factors among currently pregnant married youths in Western Oromia, Ethiopia: A mixed method
Source: PLoS One. 2021 Nov 4;16(11):e0259262. doi: 10.1371/journal.pone.0259262 (PMC8568197; doi:10.1371/journal.pone.0259262)
Supplement: S1 File — (PDF) [file pone.0259262.s001.pdf]

Appendix 1 Questionnaire (English Version)

Participant Information sheet (English Version)

Introduction: How are you, I am________________________. This is an interview to be done with you for a study that is being conducted by Wollega University, Department of public health. I would like to ask you few questions and your willingness in the study. This study is prepared to obtain relevant information on the prevalence of unintended pregnancy in Kiremu district. Your participation in the study is very important in reducing the maternal morbidity and mortality rate, which is caused by unintended pregnancy.

Study title: Unintended pregnancy and associated factors among currently pregnant married aged 15-24 years women in Kiremu district, west Ethiopia, April, 2019.

Purpose: To assess the level of unintended pregnancy and associated factors. The information you provide here will be very helpful to the investigator of this study to write a research paper for the requirement in completion of master's program. The findings of this project could help in designing priority intervention strategies for unintended pregnancy among 15-24 age groups.
Procedures: There are questions that assess associated factors for the unintended pregnancy among currently married women of 15-24 age groups. I would like to ask you to give your genuine and honest answers on the questions forwarded. If you need clarification please ask me. It will take you about 15- 20 minutes to finish this survey.
Benefits and Risks: By participating in this study and answering our questions, you will not receive any direct benefit. However, the information will help the researcher to understand factors associated for unintended pregnancy among currently married 15-24 years women of reproductive age group in order to appropriately identify future interventions related to problem to be found. Your participation in this study will not involve any risks. If a question makes you feel uncomfortable, you may choose not to answer.

Confidentiality: you will not be asked your name on to be written the survey questions. All the information you give to us will be kept private. Whatever information you provide will be kept strictly confidential. The information you give will kept in a locked file cabinet.

Only the researcher will have access to see the answers you give. No information identifying you will ever be released to anyone outside of this data collection activity.

Consent of the participant:

Could I have your permission to continue?

1. Yes

2. No stop the interview and thank the respondent. Informed consent certified by:

Interviewer: Code________________Name_______________Signature_________________

Name of data collector_________________Date________________Signature_______________

Checked by supervisor Name_________________Signature_____________Date____________

Principal investigator: Jaleta Merga Mobile: Tel: +25117852858 E-mail:jaleta2018@gmail.com

WOLLEGA UNIVERSITY DEPARTMENT OF PUBLIC HEALTH

MASTER OF REPRODUCTIVE HEALTH

PARTICIPANTS STRUCTURED QUESTIONNAIRE

Keble ____

Interviewer code -------------------/---------------------/ name----------------

Household number--------------------

Date of interview in Ethiopian calendar---------/---------/------time

Checked by, supervisor. Name ___________________________Signature: ________________

Date_________ month__________ year 2019. Starting time ____: ____

Currently pregnant married 15-24yr group women should be asked; you should circle among the multiple choices the right answers or write the code.

PART I. DEMOGRAPHIC, SOCIOECONOMIC AND CULTURAL CHARACTERISTICS

No	Questions	Choice of answers	Code	Skip to Q
100	Residence	1.Urban<br>2.Rural		
101	Age of respondent	____________ (Age in year)		
102	Religion	1.Orthodox<br>2.Muslim<br>3.Protestant<br>4.Others		
103	Educational status of the respondent	1.Cannot read and write<br>2.1-4<br>3.5-8<br>4.9-12<br>5.College and above		
104	Educational status of the husband	1.Cannot read and write<br>2.1-4<br>3.5-8<br>4.9-12<br>5.College and above		

105	What is your occupation?	1. Student<br>2.Farmer<br>3. Gov. employee<br>4.Daily laborer<br>5.Unemployed<br>6. Others		
106	What type of occupation your husband is participated in?	1.Student<br>2.Farmer<br>3.Merchant<br>4.Daily laborer<br>5.Government employee<br>6.Others		
107	What is your ethnicity?	1.Oromo<br>2.Amhara<br>3.Tigre<br>4.Others		
108	What is your average monthly income?	/_________________________/		
109	Currently whom are you living with?	1.With both parents<br>2.With mother only<br>3.With father only<br>4.With relatives<br>5.Husband<br>6.With peers/friends<br>7.Others		
110	What travel time does it take from your home to health facilities?	1.<30min<br>2.30-60min<br>3.>=60min		
111	Who was the decision maker about your health care?	1.Her self<br>2.Her husband<br>3.Huaband’s father<br>4.Husband’s mother<br>5.Other		
112	Do you have electronic media device in your home?	1.Yes<br>2.No		
113	Do you used an electronic media in the past 12 months prior to this study for any purpose?	1.Yes<br>2.No		

Part II Reproductive and health service related factors

No	Questions	Choice of answer	Code	Skip to Q No
201	Have you ever heard family planning methods?	1.Yes<br>2.No		If no stop here
202	If yes from where did you hear?	1.Radio<br>2.TV<br>3.News paper<br>4.HEWs<br>5.Health professionals<br>6.Neighbor<br>7.Friend<br>8.Husband<br>9.Others		
203	Did you discuss with your husband on modern contraceptive methods?	1.Yes<br>2.No		
204	Which methods of family planning/contraception do you?	1.Pills<br>2.Implants<br>3.Injectables<br>4.IUCD<br>5.Steralization<br>6.Others<br>7.Idon't know		
205	From where do you mostly get information on family planning methods?	1.Husband<br>2.Friends<br>3.Health extension worker<br>4.School<br>5.Mass media(TV, Radio)<br>6.Others<br>7.Idon't		

206	Do you know the place where modern contraceptive could be obtained?<br><br>(know the source of FP)	1.Yes<br><br>2.No		
207	Where you can access family planning services/contraception?	1.Governmental organization<br><br>2.Non-governmental organization<br><br>3.Health posts<br><br>4.Pharmacy<br><br>5.Others		
208	What are the advantages of modern contraceptive methods?	1.To avoid unwanted pregnancy<br><br>2.To delay mistimed pregnancy<br><br>3.To prevent STDs<br><br>4.Others<br><br>5.Idon't know		
209	Have you ever heard of emergency contraceptives?	1.Yes<br><br>2.No		
210	If yes to Q no209 Have you ever used emergency contraceptive?	1.Yes<br><br>2.No		
211	If no to Q no 210 Why did not use emergency contraceptive?	1.Health workers unwilling<br><br>2.Drugs unavailable<br><br>3.Don't know from where to get<br><br>4.Fear of social stigma<br><br>5.Health institution is far to get<br><br>6.Others		
212	Have you ever used any type of contraceptives?	1.Yes<br><br>2.No		If no skip to<br><br>Q no 215

213	If yes, which among these methods have you used?	1.Pills<br>2.Injectables<br>3.Implants<br>4.IUCD<br>5.Others		
214	What are your reasons to use contraceptives?	1.Want to improve their own health and child<br>2.Want to give birth space<br>3.Prevent unwanted pregnancy<br>4.Prevent STDs<br>5. Others		
215	If you were not using any contraceptives method to delay or avoid pregnancy, why?	1.Still want to have children<br>2.Fear of side effects<br>3.Religious reasons<br>4.Lack of knowledge of contraception<br>5.Husband/Parents opposition<br>6.Difficulty to get the method<br>7.Others		
216	Have you ever been pregnant	1.Yes<br>2.No		
217	If yes to Q 216, how many pregnancies?	/_____________/		
218	At what age did you first got married?	/___________/		
219	How old were you when you first become pregnant?	1.<18 years<br>2.>=18 years		
220	How many living children do you have now?	/_____________/		

221	The average interval between births?	/______________/		
222	"Have you intended/planned to be pregnant for this one?"	1.Yes<br>2.No		If yes to Q 224 stop here.
223	If no to question 224?	1.It is unwanted<br>2.it is mistimed		
224	If your current pregnancy was not intentional, how it happened? What was the reason?	1.Method fail<br>2.Not using FP<br>3.Forced by husband to be pregnant<br>4.Forced by mother-in-law<br>5.Fear of husband's family<br>6.Others		
225	Have you visited by health care providers (HW/HEW)?	1.Yes<br>2. No		
226	Have you had previous history of unintended pregnancy?	1.Yes<br>2.No		
227	Have you ever experienced an abortion?	1.Yes<br>2. No		
228	If yes, was the abortion induced or spontaneous?	1.Induced<br>2.Spontaneous		
229	Where it was done?	1.Public facility<br>2.Private clinic<br>3.Buying drug from pharmacy<br>4.Traditional healers<br>5.Others		

Appendix 2 Questionnaire (Afan Oromo Version)

ODEEFFANNOO NAMA HIRMAATUUF KEENNAMU

Seensa

Kabajamoo Hirmaataa: Maqaan Koo ______________________ jedhama.

Du'aatii fi Miidhama haadholii fi daa'immanii xiqqeessuuf akkasumas tajaajila fayyaa haadholii fi daa'immanii fooyyessuuf rakkolee dhimma **ulfa hin karoorfamiinii fi sababoota kana waliin wal qabatanii jiran gad fageenyaan qoratanii hubachuun barbaachisaadha**.

Mata duree qorannoo: ulfa hin karoorfamiinii fi sababoota isaa aanaa Kiiramuu, godina wallaggaa bahaa, dhiha Itoophiyyaatti, Caamsaa, 2019.

Kaayyoo: kaayyoo qorannoo kanaa Sababoota ulfa hin karoorfamiinii wajjin wal-qabatan adda baasuu dha. Kanaaf odeeffannoon isin laattan kun Qorataa kanaaf waraqaa qorannoo Guutuu ta'eebarreessee dhiheessuu fi sagantaa Digirii 2^{ffaa}isaa xumuruuf iddoo olaanaa qaba. Bu'aan qoraannoo kanaa qaamota Sagantaa Tajaajila fayyaa maatii irratti hojjetaniif akkaa galtee tokkootti ta'uun kara fuulduratti caalmaatti irratti karoorfatanii hojjechuuf gargara.

Haala deemsaa isaa: Sababoota ulfa hin karoorfamiin dubartoota reefuu heerumaanii umurii da'uumsaa keessattii argaman wajjin walqabatan addaa baasuuf gaaffiiwwaan qopha'aan jiru. Kanaaf waantaan isiin gaafachuu barbaadu gaaffii gaafadhuuf amanttaa guutuu ta'een deebii quubsaa akka naa keennitan kabajaanan isin gaafadha.Yoo ibsa dabalataa barbaaddan na gaaffachuu dandeessu. Gaaffii fi deebiin keenya kun kan inni itti xumuramuu danda'uu daqiiqaa 20-25 gidduutti dha.

Faayidaalee fi miidhaalee isaa: sababa qorannoo kana keessatti deebii deebisuun hirmaattaniif faayidaa isin kallattiin argattan hin jiru. Haa ta’uu malee odeeffannoon isin laattan qoratichi rakkoolee ulfa hin karoorfamiinii fi sababoota isaan wajjin wal qabatu sirriitti addaa baasuun kallattii gara fuulduraa ka’uuf ni gargara. Hirmaannaa qorannoo kana keessatti gootaniif rakkoon isinirraga’uu hin jiru. Yoo gaaffiin gaafatamtan isinitti toluu baate deebii laachuu dhiisuu nidandeessu .

Iciitii: gaafannoo qophaa’e irratti maqaa keessan barreessuun hin barbaachisu. Garuu odeeffannoon isin nuu laattan hundi isaa icciitiin isaa seeraan ni qabamu. Odeeffannoon isiin nuu lattaan kun qoraatan qofti dhimma qorannoo kanaaf qofaa deebii isin laattan qofaa itti fayyadama. Odeeffannoon isin laattan kamiyyuu qaama odeeffannoo funaanu ala nama biraatti dabarsamee hin laatamu.

Kanaafuu Qorannoo kana keessatti hirmaachuuf eyyamamoo dhaa? 1. Eyyeen 2. Lakkii

Unka Walii galtee

Hirmaachuuf itti walii galuu **Eeyyee {}Lakki {}**
Koodii Hirmaataa ___________Guyyaa___________Mallattoo__________

Maqaa fi ragaa Odeeffannoo sassaabaa____________________Guyyaa__________Mallattoo______

Kan mirkaneesse: Maqaa to’ataa____________Mallattoo___________Guyyaa___________

Odeeffannoo fi Gaaffii dabalataa dhimma qorannoo kana irratti yoo qabaattan teessoo armaan gadiin nu qunnamuu dandeessu.

Qorataa jalqabaa: Jaallataa Margaa

Mobile:Tel:+25117852858 E-mail:jaleta2018@gmail.com

GAAFANNOO BARREEFFAMAA(BAR-GAAFFII) HIRMMAATTOTA QORANNOO

Ganda ___________________________________

Kooddii nama gaafatamuu __________________

Lakk mana (CHIS irraa) ____________________

Guyyaa gaaffiin Gaggeeffame _______ / ________/ 2011

Nama Gaafate Maqaa _____________________Mallattoo _______ Guyyaa _________

To'ataa Maqaa _____________________ Mallattoo _______ Guyyaa _________

Dubartoota ulfaa Umurii 15-24 keessa jiraatanii bultii ijaarratanii/heerumanii jirantu gaafatama. Filannoowwan tarreeffamanii jiran keessaa Kan deebbii ta'etti mari ykn immoo tarree kooddii jedhu keessatti kooddii deebbii deebisanii barreessi.Gaaffilee filannoo hin qabne deebbii hirmaatonni deebisantu barreeffama.

Sa'aatii itti eegalame _____:_____

KUTAA 1[FFAA] GAAFFILEE DIMOOGIRAAFII, HAWWAAS DINAGDEE FI DUDHAALEE

**Lakk**	**Gaaffilee**	**Filannoo deebii**	**Koodii03**	**Gara gaaffii __tti ce'i.**
100	Bakka Jireenyaa	1.Magaalaa<br>2.Baadiyyaa		
101	Umurii Hirmaattuu	Waggaa dhaan galchi______		
102	Umuriin itti Jalqaba Gaa'ela dhaabbatte meeqadha?	/__________________/		
103	Amantii Hirmaattuu	1.Kiristaana<br>2.Musilimaa<br>3.Pirootestaantii<br>4.Kanneen biroo		

104	Sadarkaa Barnootaa	1.Dubbisuu fi barreessuu kan hin dandeenye<br>2.1-4<br>3.5-8<br>4.Koollejjii		
105	Sadarkaa Barnootaa A/warraa	1.Dubbisuu fi barreessuu kan hin dandeenye<br>2.1-4<br>3.5-8<br>4.9-12<br>5.Koollejjii		
106	Gosa Hojii Hirmaattuu	1. Barattuu<br>2. Qonnaan Bultuu<br>3. H/Mootummaa<br>4. Dafqaan Bultuu<br>5. Hojii kan hin qabne<br>6.Kan biroo		
107	Gosa Hojii abbaa warraa	1.Baratаa<br>2.Qonnaan Bulaa<br>3.Daldalaa<br>4.Dafqaan bulaa<br>5.Hojjetaa Mootummaa<br>6.Kan biroo		
108	Sabummaa Hirmaattuu	1.Oromoo<br>2.Amaaraa<br>3.Tigiree<br>4. Kan biroo		
109	Galiin keessan ji'aa giddu galeessaan meeqa?	/____________/		
110	Yeroo ammaa Eenyu waliin Jiraatta?	1.Abbaa fi Haadha waliin<br>2.Haadha Qofa		

		3.Abbaa Qofa<br>4.Fira waliin<br>5.Abbaa warraa waliin<br>6.Hiriyyaa dhiiraa waliin<br>7.Kan biroo		
111	Dhaabanni fayyaa mana jireenyaa keessan irraa Yeroo hammam fudhata?	1.<30min<br>2.30-60min<br>3.>=60min		
112	Kunuunsa tajaajila fayyaa kee irratti kan murteessu Eenyu?	1.Haadha warraa<br>2.Abbaa warraa<br>3.Abiyyuu<br>4.Amaatii<br>5.Kan biroo		
113	Mana keessan keessaa raadiyoo/Televizinii qabdu?	1.Eeyyee<br>2.Miti		
114	Ji’oota 12 dura meeshaalee miidiyaa fayyadamtanii beektuu?	1.Eyyee<br>2. Miti		

Kutaa: 2ffaa Gaaffilee Fayyaa wal hormaataa fi Kenna Tajaajila fayyaa

201	Waa'ee Maloota karoora maatii Dhageessee beektaa?	1.Eyyee<br>2. Miti		Yoo Lakki ta'e asumatti dhaabi
202	Yoo gaaffiin 201 eeyyee ta'e Eessaa Dhageessee beektaa?	1.Raadiyoo<br>2.Televeezinii<br>3.Barruulee<br>4.Ekisteeshinii fayyaa<br>5.Ogeessa fayyaa<br>6.Ollaa<br>7.Hiriyyaa<br>8.Abbaa warraa<br>9.Kanneen biroo		
203	Waa'ee karoora maatii ilaalchisee abbaa warraa kee waliin ni mari'attuu?	1.Eeyyee<br>2.Miti		
204	Gosoota karoora maatii armaan gadii keessa kam beekta?	1.Piilsii<br>2.Kan gogaa irree keessan kennamu<br>3.Diipoo<br>4.Luuppii<br>5.Mala karoora maatii dhaabbata<br>6.Kanneen biroo		
205	Odeeffannoo Waa'ee karoora maatii Yeroo baayyee Eessaa argattuu?	1.Abbaa warraa<br>2.Hiriyyaa<br>3. Dhaabbata fayyaa.<br>4.Mana Barumsaa<br>5.Miidiyaa<br>6.Kannneen biroo		

206	Iddoo itti tajaajilli karoora maatii ammayyaa argamu ni beektaa?	1.Eeyyee<br>2.Miti		
207	Gaaffiin 206 eeyyee yoo ta’e Eessaa argattuu?	1.Dhaabbata fayyaa Mootummaa<br>2.Dhaabbata fayyaa miti Mootummaa<br>3.Keellaa fayyaa<br>4.Faarmaasii<br>5.Kanneen biroo		
208	Faayidaan Maloota karoora maatii ammayyaa maal fa’i?	1.Ulfa hin barbaadamnne ittisuuf<br>2.Ulfa Yeroo malee dhorkuuf<br>3.Dhukkkuboota wal qunnamtii saalaa ittisuuf<br>4.Kan biroo		
209	Mala karoora maatii Yeroo hatattamaa Dhageessee beektaa?	1.Eeyyee<br>2.Miti		
210	Yoo gaaffiin 209 eeyyee ta’e mala karoora maatii Yeroo hatattamaa fayyadamtee beektaa?	1.Eeyyee<br>2.Miti		
211	Yoo gaaffiin 210 miti ta’e sababa maaliif fayyadamtee hin beektu?	1.Ogeessonni fayyaa fedhii dhabuu<br>2.Qorichi sababa dhibeef<br>3.Iddoo inni itti argamu beekuu dhabuu<br>4.Loogii hawaasaa sodaachuu<br>5.Dhaabanni fayyaa sababa fagoo ta’eef<br>6.Kan biroo		

212	Maloota karoora maatii ammayyaa fayyadamtee beektaa?	1.Eeyyee<br>2.Miti		Yoo Lakki ta'e gara<br>Gaaffii 215 itti ce'i
213	Yoo eeyyee ta'e gosa kam fayyadamtee beekta?	1.Piilsii<br>2.Diipoo<br>3.Kan gogaa irree jalaan kennamu<br>4.Luuppii<br>5.Kanneen biroo		
214	Maloota karoora maatii sababa maaliif fayyadamtuu?	1.Fayyaa ofii fi kan daa'imaa fooyyessuuf<br>2.Walirraa fageessanii da'uuf<br>3.Ulfa hin barbaadamnne ittisuuf<br>4.Dhukkuboota wal qunnamtii saalaan daddaraban ittisuuf<br>5.Kanneen biroo		
215	Maloota karoora maatii hin fayyadamne yoo ta'e sababa maaliif?	1.Daa'ima waanan barbaadeef<br>2.Miidhaa cinaa inni qabu sodaachuun<br>3.Sababa amantiinkoo na dhorkuuf<br>4.Waa'ee karoora maatii ilaalchisee beekumsa waanan hin qabnef<br>5.Abbaan warraako/Maatiin waan na dhorkaniif<br>6.Argachuu waanan hin dandeenyeef<br>7.Kanneen biroo		
216	Kanaan dura ulfa taaatee beektaa?	1.Eeyyee<br>2.Miti		

217	Yoo gaaffiin 216 eeyyee ta’e ulfikee Yeroo ammaa kun meeqaffaadha?	/___________________/		
218	Umuriin itti Jalqaba ulfa taate meeqadha?	1.<18<br>2.>=18		
219	Yeroo ammaa Daa’imman meeqa qabdu?	/___________________/		
220	Giddu galeessaan hangam walirraa fageessitanii deessu?	/_____________________/		
221	Ulfa Yeroo ammaa kana karoorumaan godhattanii?	1.Eeyyee<br>2.Miti		
222	Gaaffiin 221^{ffaa} miti yoo ta’e	1.Waanan hin barbaadnneef<br>2.Yeroo malee waan ta’eef		
223	Yoo gaaffiin 221^{ffaa}miti ta’e sababa maaliif(akkamitti) ta’e	1.Rakkoo qorichaa<br>2.Karoora maatii fayyadamuu dhiisuun<br>3.Dhiibbaa abbaa warraakoorraa kan ka’e<br>3.Dhiibbaa amaatiikoorraa kan ka’e<br>4.Maatii abbaa warraako sodachuun<br>5.Kan biroo		
224	Ulfa ta’uu keessaniin dura ogeessa fayyaan ilaalamtanii beektuu?	1.Eeyyee<br>2.Miti		
225	Kanaan duraa Ulfi karooraan alaa si mudaatee beekaa?	1.Lakki<br>2.Miti		
226	Ulfa of irraa baasuun si qunnamee beekaa?	1.Eeyyee<br>2.Miti		

227	Yoo gaaffiin 229 eeyyee ta’e gosti isaa maali?	1.Gochaa ofiin kan raawwate<br>2.Ofiin kan raawwate		
228	Ulfi si irraa ba’e kun eessatti?	1.Dhaabbata fayyaa Mootummaa<br>2.Kilinikaa dhuunfaa<br>3.Kuusaa qorichaa<br>4.Namoota aadaa biratti		
