# Supplementary material for: Unintended pregnancy and the factors among currently pregnant married youths in Western Oromia, Ethiopia: A mixed method
Source: PLoS One. 2021 Nov 4;16(11):e0259262. doi: 10.1371/journal.pone.0259262 (PMC8568197; doi:10.1371/journal.pone.0259262)
Supplement: S2 File — (PDF) [file pone.0259262.s002.pdf]

Key Informant Interview Guide (English Version)

We are interested in knowing the level of unintended pregnancy and associated factors among currently pregnant married women 15-24yr in this district. We will ask you some important question that will help in assessing the level and associated factors for unintended pregnancy. Here is the guiding check list this will take few minutes and as much as possible the real information is needed for accuracy of this study which in turn to reduce maternal mortality rate induced by unintended pregnancy.

Name of Kebele ________________________________

Code ____

1. What are problems in young women in related with unwanted/mistimed pregnancy?

    [Any problems phased including complications]

2. Why young women's are facing the problems of unintended pregnancy in this kebele?
3. What are the socio-cultural factors associated with unintended pregnancies among young women?
4. What are strategies you used to aware about Family Planning service in the community?
5. Attitude of the community to ward family planning in the kebele.
6. What are the problems you encountered in providing Family Planning service for currently married young women in the community?

Afan Oromo version Key Informant Interview Guide

Gaaffii Afaaniffaa (Af- Gaaffii) Hirmaattota Filatamaniif Qophaa'e

Kuni kan qophaa'eef akka Aanaa kanaatti sadarkaa ulfi karooraan alaa fi sababoota isaanii jedhu irratti dubartoota reefuu heerumanii umuriin isaanii waggaa 15-24 gidduu jiran beekuuf fedha godhanneeti. Haaluma kanaan sadarkaa fi sababoota rakkoo kanaa xiinxaluuf kan nu gargaaran gaaffileen murteessoo ta'an is in gaafanna. Cheeklistiin kuni daqiiqaa muraasa qofa kan fudhatuufii hanga danda'ametti odeefannoon sirrii fi dhugaa irratti hundaa'e barbaachisaadha. Sababni isaa qorannoon kuni du'aatii haadholii sababa ulfa karooraan alaan dhufu xiqqeessuu keessatti gahee olaanaa qaba.

Maqaa Gandaa______________________________

Kooddii____________________________________

1. Akka Naannoo Keessaniitti Rakkooleen ulfa karooraan alaa waliin wal qabatee Dubartoota reefuu heeruman irratti mul'atu maal fakkaata?
   (**Miidhaa isaa dabalatee**)
2. Akka ganda keessaniitti rakkoon kun dubartoota reefuu heeruman maaliif isaan qunnama?
3. Akka naannoo keessaniitti dhiibbaa Aadaan dubartoota reefuu heeruman irraan geessisaa jiru maali dha?
4. Akka ganda keessaniitti maloonni isin itti fayyadama karoora maatii hawaasa hubachiisuuf fayyadamtan maal faadha?
5. Ilaalchi hawaasnni ganda keessanii karoora maatii irratti qabu maalidha?
6. Rakkooleen Tajaajila karoora maatii dubartoota reefuu heeruman biratti isin qunnamaa jiru maalifaadha?
